# Preclinical Alzheimer disease: identification of cases at risk among cognitively intact older individuals

**DOI:** 10.1186/1741-7015-10-127

**Published:** 2012-10-25

**Authors:** Maciej J Lazarczyk, Patrick R Hof, Constantin Bouras, Panteleimon Giannakopoulos

**Affiliations:** 1 Department of Mental Health and Psychiatry, University Hospitals of Geneva and Faculty of Medicine of the University of Geneva, 1225 Geneva, Switzerland; 2 Fishberg Department of Neuroscience and Friedman Brain Institute, Mount Sinai School of Medicine, New York, NY 10029, USA

**Keywords:** Alzheimer disease, asymptomatic, cerebral amyloidosis, cognition, compensatory phenomena, dementia

## Abstract

Since the first description of the case of Auguste Deter, presented in Tübingen in 1906 by Alois Alzheimer, there has been an exponential increase in our knowledge of the neuropathological, cellular, and molecular foundation of Alzheimer's disease (AD). The concept of AD pathogenesis has evolved from a static, binary view discriminating cognitive normality from dementia, towards a dynamic view that considers AD pathology as a long-lasting morbid process that takes place progressively over years, or even decades, before the first symptoms become apparent, and thus operating in a continuum between the two aforementioned extreme states. Several biomarkers have been proposed to predict AD-related cognitive decline, initially in cases with mild cognitive impairment, and more recently in cognitively intact individuals. These early markers define at-risk individuals thought to be in the preclinical phase of AD. However, the clinical relevance of this preclinical phase remains controversial. The fate of such individuals, who are cognitively intact, but positive for some early AD biomarkers, is currently uncertain at best. In this report, we advocate the point of view that although most of these preclinical cases will evolve to clinically overt AD, some appear to have efficient compensatory mechanisms and virtually never develop dementia. We critically review the currently available early AD markers, discuss their clinical relevance, and propose a novel classification of preclinical AD, designating these non-progressing cases as 'stable asymptomatic cerebral amyloidosis'.

## Introduction

In 1906, Alois Alzheimer documented the case of Auguste Deter, a patient with a combination of cognitive deficits, psychiatric symptoms, and macroscopic and microscopic brain lesions [1,2]. This histopathological and clinical constellation was first designated by Emil Kraepelin as Alzheimer's disease (AD), and later on as dementia of the Alzheimer-type (AD-type dementia). Since this first definition, an impressively broad spectrum of mechanisms have emerged, including genetic vulnerability, and the molecular, cellular, and neurochemical abnormalities closely related to AD pathogenesis [3-5]. Some examples illustrate the diversity of the field and the difficulty in formulating and following up a unique causal hypothesis for such a heterogeneous disorder. Initially, abnormal protein filaments were described structurally in amyloid plaques (APs) and neurofibrillary tangles (NFTs) [6,7], and more than 200 large clinicopathological studies in hospital-based and community-based series have shown the differential effects of fibrillar amyloid deposits and NFT formation on cognitive performances across the age spectrum [8-11]. Following the pioneering observations of Tomlinson and coworkers, which indicated the presence of substantial AD lesion densities in cognitively intact older people [12], the systematic work of Braak and collaborators showed the stepwise progression of amyloid deposits and NFTs in brain aging and AD [13,14]. Amyloidogenic fragments (monomers, dimers, oligomers) were soon purified from AD-affected brains, and tau protein was identified as the main constituent of NFT [15-17]. Yankner and coworkers then identified the neurotoxic properties of the amyloid beta (Aβ) protein [18]. In the 1970s, the cholinergic hypothesis of AD emerged and growing interest was raised with the identification of the first therapeutic targets for drug development [19-21]. In the early 1980s, medial temporal lobe subdivisions became the focus of interest, following the detailed description of atrophy patterns in association with progressive memory loss in mild and prodromal forms of AD [22-25]. In the early 1990s, the first genes conferring a risk for early-onset (amyloid beta (A4) precursor protein (APP) and presenilin (PSEN)1 and 2) and late-onset (apoliprotein (APO)ε4) AD were identified [26-29]. Recently, these discoveries have been followed by identification of polymorphisms in other genes, probably involved in Aβ processing and clearance. Large genome-wide studies have identified associations between late-onset AD and polymorphisms in the genes *clusterin*, *CR1 *(complement receptor 1), *SORCS1 *(sortilin-related VPS10 domain containing receptor 1) and *PICALM *(phosphatidylinositol binding clathrin assembly protein) [30-32], observations that were subsequently confirmed by other groups in diverse ethnic cohorts [33-40]. Stemming from these milestones in the understanding of AD pathology, the past decade saw the development of animal models and clinical trials with immunization-based therapeutic strategies [41-49]. Despite these efforts, numerous crucial questions remain unanswered. Why are only some brain regions and neuronal types preferentially affected? Why, despite the presence of Aβ deposits, do some individuals not present clinically overt dementia? Is there any natural compensatory mechanism(s) that might counterbalance the toxic effect of Aβ? Is AD an age- or aging-related pathology?

The major recent conceptual evolution has been the conversion from a 'static and defensive' view of AD pathogenesis to one that is 'dynamic and compensatory'. According to the first model, AD lesions chronically attack the human brain, leading to synaptic and neuron loss before cognitive breakdown. Whether and when this occurs depends mainly on the severity of the external aggression and on the structural reserve [50-52]. The second model suggests that the clinical expression of the disease may vary widely over time, depending on individual vulnerability to the initial phases of the degenerative process, the severity of the AD pathological process at the molecular and cellular levels, and the efficiency and evolution over time of compensatory brain mechanisms.

According to this dynamic model, future curative treatments should be administrated long before the emergence of clinically overt symptoms, either to counterbalance the biological compromise that precedes the cognitive breakdown or to promote functional compensation [53]. The limited therapeutic efficacy of the first vaccination trials in moderate AD may have reflected the irreversible brain damage that had already taken place in these cases. This is also supported by some data from animal models, which showed that the efficacy of β-amyloid_1-42 _(Aβ42) immunization was largely reduced in mice with significant amyloid deposition [54]. In line with these findings, clinical trials using acetylcholinesterase inhibitors in patients with mild cognitive impairment (MCI) all failed to show any clear benefit [55,56]. In fact, more recent evidence has shown that all of the major pathophysiological processes associated with AD have already occurred by the time MCI is diangosed, introducing the notion that patients with clinically early AD may display substantial biological deficits [57-62]. Consquently, in order to set up true secondary prevention in AD, it is crucial to identify cognitively intact individuals at risk for AD, working on the assumption that some objectively measurable AD markers exist that precede clinical symptoms by several years and define a stable 'pre-AD' stage.

### Preclinical Alzheimer disease

AD was perceived for the first time more as a dynamic process than a stationary state in the late 1980s, and the idea that the pathological process begins long before clinical symptoms become apparent has gained increasing interest [63]. Even though normal brain aging and AD-type dementia are both associated with loss of neurons and accumulation of APs and NFTs, the extent and distribution of the lesions is not the same in both case [51,52,63]. In non-demented older individuals, NFTs are mainly found in the hippocampus, whereas in the course of dementia a progressive spread of NFTs into the temporal neocortex is seen. It has been shown that the total NFT counts in the hippocampus, entorhinal cortex and prefrontal area 9 is strongly predictive of cognitive status [9,64]. Moreover, the neuron loss and its spatial distribution in normal aging is also qualitatively and quantitatively different from that in AD, where a massive loss of pyramidal neurons takes place mainly in the cornu ammonis (CA)1 field of the hippocampus [9,65-67]. The differences between normal aging and AD were recently clarified and formalized by Dubois and collegues, who proposed a novel classification of AD, which distinguishes three stages of the disease: preclinical AD, prodromal AD (equivalent of MCI), and dementia [68] In this review, we focus on preclinical AD cases by addressing the clinical relevance of biomarkers that could predict their cognitive evolution.

### Biomarkers of preclinical Alzheimer disease

#### CSF markers

Even though a definite diagnosis of AD can be formulated only neuropathologically, CSF markers play an important supportive role in the clinical diagnosis of probable AD [68]. The levels of Aβ42 in the cerebrospinal fluid (CSF) are inversely correlated with AP burden, and the CSF tau levels reflect the progression of tau-related pathology within the cerebral cortex [69]. Low levels of Aβ42, together with increased levels of phosphorylated (p)-tau and total (t)-tau, identify AD with good accuracy, and can be useful in the differential diagnosis of dementia [70-73]. However, these markers are not specific for dementia. Low levels of Aβ42 appear early in the course of AD, and have been shown to predict conversion from MCI to AD [74]. Other authors have shown that abnormalities in CSF levels of Aβ42 and tau can be detected even earlier, in people who are still cognitively normal (CN), preceding MCI by several years [75-83].

Low CSF Aβ42 levels in CN older adults correlate with whole-brain volume [76], atrophy rate [66], and cortical amyloid load [75,77]. CN carriers of the APOε4 allele, which confers a risk for late-onset AD, and is associated with slightly lower cognitive function in adulthood [84], also have lower CSF Aβ42 levels [83,85]. Contrastingly, an increase in CSF tau and p-tau in cognitively intact individuals correlates with cortical amyloid load [75] and cerebral hypometabolism in the posterior cingulate, precuneus, and parahippocampal regions [79]. Interestingly, a high CSF tau:Aβ42 ratio in CN adults is related to cortical lesions and pathological changes in the white-matter microstructure, which probably precede structural alterations in the cortex [83,86].

The exact timing of the appearance of these CSF markers is still a matter of debate. Even though it seems that a decrease in CSF Aβ42 concentrations precedes elevation of tau levels [75], both parameters can be considered as early hallmarks of AD. Reduction in CSF Aβ42 levels was shown to precede cognitive decline in non-demented subjects for as long as 8 years, and a combination of CSF Aβ42 and p-tau might further increase its sensitivity and specificity in prediction of dementia [82,87]. Indeed, high CSF tau:Aβ42 and p-tau:Aβ42 ratios were shown to be a powerful predictive factor for the conversion of normal cognition to dementia, preceding the conversion by years [77,80-82]. These observations are further supported by independent studies of familial AD, in which decreased levels of Aβ42 and increased levels of tau and p-tau in the CSF were found in asymptomatic carriers of *PSEN1 *and *APP *pathogenic mutations, more than 10 years before the clinical onset of the disease [88-90].

#### Positron emission tomography with Pittsburgh compound B

Positron emission tomography (PET) imaging of the amyloid-binding agent Pittsburgh compound B (PET-PiB) allows for semiquantitative *in vivo *analysis of the brain Aβ load and its spatial distribution. Like CSF Aβ42 levels, PET-PiB is a valuable marker in the differential diagnosis of dementia [91]. It is closely correlated with amyloid plaque burden at autopsy [92], and is inversely related to CSF Aβ42 levels [75,77,93]. However, it is not specific to dementia; up to 20% of CN people have a considerable PiB load in the brain, falling into a 'PiB-positive' category [91,94-97]. However, though still within the normal cognitive range, these PiB-positive controls have slightly lower cognitive performance compared with PiB-negative people [98]. They have a very subtle episodic memory impairment [96,99], smaller hippocampus volume [99], and accelerated rate of cortical atrophy [100]. The conversion from a PiB-negative to a PiB-positive state reflects a very early step in AD development [95]. These PiB-positive individuals clearly represent a subpopulation at risk for dementia [93,101,102]. For instance, there is a higher prevalence of PiB positivity among CN subjects with known genetic AD risk factors, and CN carriers of APOε4 have an increased incidence rate of conversion from PiB-negative to PiB-positive status, many years before the clinical onset of AD [95]. Similarly, asymptomatic carriers of pathogenic *PSEN1 *or *APP *mutations, responsible for early-onset AD, have increased PiB retention in the cortex and striatum [103-105]. Together, these data support the idea that increased PiB load may serve as a predictive factor of AD-type dementia in healthy older individuals [100,106,107]. Whether measurement of PET-PiB levels is a better predictive factor than CSF Aβ42 levels remains unclear [75,108-111].

Individual risk estimation solely on the basis of PiB status remains difficult because many CN individuals have a brain PiB load practically indistinguishable from patients with overt dementia [101]. These 'PiB-high' subjects have a more rapid increase in PiB brain load over time than do PiB-positive individuals with relatively lower PiB signal, and are thought to be at higher risk for AD-type dementia than 'PiB-low' individuals [107,112]. However, not all 'PiB-high' individuals evolve to dementia; in longitudinal studies, some remained CN for at least 4 years [107]. Moreover, even in cases of monozygotic twins with increased PiB load, cognitive discordance (one twin demented and the other one CN) has been described [113], suggesting that environmental and epigenetic factors modulate the effects of Aβ on cognition.

#### Fluro-D-glucose positron emission tomography

PET imaging with 2-deoxy-2[^18^F]fluoro-D-glucose as a tracer (FDG-PET) measures cerebral glucose metabolism, which reflects the level of synaptic activity. Perturbations in glucose metabolism have been repeatedly reported in AD [114-117]. In order to investigate whether the synaptic dysfunction seen with FDG-PET precedes the clinical symptoms in AD, numerous studies have been performed in CN individuals at risk of AD, all of which documented hypometabolism in the regions typically affected in AD [118-131]. A substantial reduction in glucose metabolism in the posterior cingulate, precuneus, parietal, and prefrontal cortex was shown in middle-aged CN carriers of the APOε4 allele [120,124], and this observation was recently reproduced in Latino populations [123]. A gene-dosage effect was documented in this context, with a more pronounced reduction in glucose metabolism in CN APOε4 homozygotes than in heterozygotes [122]. Interestingly, this brain hypometabolism in APOε4 carriers is a gradually progressing process that leads to a further decline after a 2-year period, as shown in follow-up studies [121,125]. It is thus likely that the brain hypometabolism in posterior cortical areas represents a valuable preclinical AD biomarker, preceding overt dementia by several years [121,125]. Confirming this viewpoint, Reiman and colleagues showed that low glucose metabolism in the posterior cingulate, parietal, temporal, and prefrontal cortex of CN APOε4 carriers can be detected as early as the third decade of life [118] preceding clinical disease onset as much as 40 to 50 years. This unexpected observation (in view of the extremely long preclinical period) is consistent with the substantial NFT formation in brains of young (less than 40 years old) CN APOε4 carriers [132].

However, the exact pathophysiological significance of the reduced cerebral glucose metabolism in CN individuals remains unclear. Although it may represent an indirect marker of cortical vulnerability to the degenerative process, it does not determine the occurrence of dementia; for instance, reduction in glucose metabolism in temporal cortex was found in cognitively discordant monozygotic twins [126,127]. The link with APOε genotype is also difficult to interpret. Even though predominantly studied in the context of APOε4 carriers, this hypometabolism seems to be an integral element of AD pathogenesis, without a strict association with a single genetic risk factor. In fact, hypometabolism in parietotemporal, posterior cingulate, and medial temporal cortex was reported in CN individuals with a family history of AD independent of their APOε genotype [129,133], and also in asymptomatic individuals carrying pathogenic mutations in the *APP *gene [130,131].

#### Structural MRI

Even though structural brain changes are usually preceded by alterations in PET and CSF markers, abnormalities in structural MRI become detectable well before the first clinical signs of the disease, and thus might serve as a marker of preclinical AD. The exact hierarchical patterns of cortical atrophy vary greatly over time, but there is broad consensus that the atrophy of the medial temporal lobe (particularly the hippocampus) and cortical thinning in certain AD-vulnerable regions are the first MRI signs of emerging AD [134-142].

In asymptomatic individuals at risk for early-onset familial AD (those carrying a pathogenic APP mutation), volumetric MRI analysis identified decreased hippocampus volume 2 to 3 years before dementia onset [143]. Other authors have reported that decreased hippocampus volume in community-based older individuals precedes dementia by as much as 6 years [134-138], which fits well with the neuropathological findings [144]. Further subregional analyses have shown that in CN subjects, the volume of restricted parts of the hippocampus (the CA1 and subiculum) is more closely associated with conversion to MCI than is the total hippocampus volume [136,139]. The volume loss in these regions precedes cognitive decline and conversion to MCI by a few years, and was able to discriminate cognitively stable from declining individuals with up to 93% accuracy, especially when combined with neurocognitive testing [136,139]. Using high-dimensional diffeomorphic transformations, Csernasky and colleagues evaluated the surface of the hippocampus, and found that inward deformation of the left hippocampal surface within the CA1 field is an early predictor of the conversion to dementia in CN older subjects [135].

Volume reduction in other medial temporal lobe subdivisions besides the hippocampus, and acceleration of ventricular volume expansion [145], have also been described in CN individuals at risk for AD [136,143,146-148]. Decreased entorrhinal cortex volume was shown to precede significant cognitive decline by 4 years and, together with hippocampus volume, to predict cognitive decline in CN subjects with an accuracy reaching 80% (up to 90% when combined with decreased hippocampus volume) [136]. Similar results were reported for the reduced volume of the anteromedial temporal cortex [146,147], the prediction accuracy of which was further improved when neuroimaging data were combined with neuropsychological testing [136,146].

Recently, early structural abnormalities in the neocortex have aroused growing interest [143,146,149,150]. Decreased gray-matter volume in the parietal lobe, notably in the angular gyrus, has been described in CN individuals in advance of MCI development [146]. Moreover, prefrontal cortex atrophy in CN individuals was found to precede dementia onset over a 6-year period, and appeared to be even a more sensitive predictive factor than hippocampal volume [149]. Dickerson *et al. *reported that the analysis of multiple regions preferentially affected in mild AD (referred to as the 'cortical AD signature') could be useful in predicting AD conversion in CN individuals [140-142]. Subtle cortical thinning in a set of seven to nine preselected neocortical regions was shown to be associated with increased risk for AD development, and it preceded loss of hippocampus volume [140,142,151]. Notably, atrophy in these regions was detectable more than 10 years before clinical onset of the disease, and correlated with the CSF Aβ42/tau ratio and amyloid load measured by PiB binding [142,150,152].

#### Functional MRI

Functional connectivity between different brain regions is disrupted early in the course of AD [153-156], possibly reflecting the deleterious effects of Aβ on synapses and glucose metabolism. At the whole-brain level, such early dysfunctions trigger multiple compensatory functional rearrangements of the neural networks to preserve cognitive performance [157-164]. Using functional (f)MRI, it was shown that in CN APOε4 carriers, the magnitude of brain activation in the parietal and prefrontal regions during memory tasks is higher than in controls, and the extent of brain activation correlates with subsequent memory decline in these subjects [157-160]. This extensive extrahippocampal activation may represent an attempt to counterbalance subtle deficits in hippocampal function, and is thought to represent an early functional sign of emerging AD in CN individuals [161]. The same kind of overactivation in the frontal and temporal lobes during memory encoding has been seen in older people at high risk for late-onset AD, independently of their APOε genotype, as much as 10 years earlier than the estimated AD onset [162]. Interestingly, such a functional reorganization is not limited to the memory-related tasks, but has been also reported in the parietal lobes during a mental rotation test [163], and in the medial temporal lobe, posterior cingulate cortex, bilateral thalamus, and caudate nucleus, during divided-attention tasks [164].

### The dynamic cascade in preclinical AD

Accumulating data on preclinical AD markers obliges us to revisit the traditional view of the degenerative process and its temporal evolution in brain aging. Jack and coworkers recently proposed such a hypothetical model, which defines ordered, sequential appearance of early markers during preclinical phase of AD [165]. According to this model, the markers related to amyloid formation, namely decreased CSF Aβ42 levels and increased PiB-PET Aβ brain load, become detectable first. Later on, the markers of synaptic dysfunction and neurodegeneration, such as abnormalities in FDG-PET and fMRI patterns, appear followed by an increase in CSF tau protein levels. At more advanced stages, structural brain changes, such as cortical atrophy and decreased hippocampus volume, can be detected by MRI. All of these markers become positive before the first signs of cognitive decline. These authors suggested that the changes in these preclinical markers gradually increase over time, probably following a sigmoid trajectory [165], an idea that has been partly confirmed by recent experimental studies [166].

This model cannot be seen as definitive, and several issues remain to be addressed. For instance, abnormal brain glucose metabolism is seen as early as the third decade of life, and is the earliest detected change in individuals at risk for late-onset AD [118]. Whether Aβ could also be detected in these subjects if sufficiently sensitive techniques were available remains unknown. Certainly, the exact order of marker appearance depends on the accuracy of the diagnostic techniques, and thus is likely to changeas new developments arise. Nevertheless, the concept surrounding this model is innovative, because it describes AD as a dynamic and biologically unstable process, rather than a stable nosological condition, and takes into account sequential marker changes during preclinical stages. In line with this model, new diagnostic research guidelines have recently been formulated, discriminating three stages of preclinical AD [167]. Stage 1 refers to asymptomatic brain amyloidosis, and is based on positive amyloid markers (PiB-PET and/or low CSF Aβ42 levels). Stage 2 encompasses brain amyloidosis accompanied by markers of neurodegeneration (abnormalities in FDG-PET/fMRI or high CSF t-tau and p-tau levels or atrophy on structural MRI). Stage 3, which refers to brain amyloidosis with signs of neurodegeneration as specified for stage 2, is accompanied by a subtle cognitive decline that does not yet fulfill the criteria for MCI. In population-based studies, 43% of CN oldersubjects had none of the early AD markers, while 16% met the criteria for stage 1, 12% for stage 2, and 3% for stage 3. Notably, 23% of subjects were not compatible with any of the stages and were defined as 'suspected non-AD pathophysiology' [97]. Interestingly, the transition through these preclinical stages (stage 1 to stage 2 to stage 3) was associated with an increased risk of conversion to MCI or dementia [168], suggesting that this classification adequately reflects the natural course of the disease.

### Presymptomatic or asymptomatic Alzheimer disease: what exactly do we detect?

**Figure 1:**
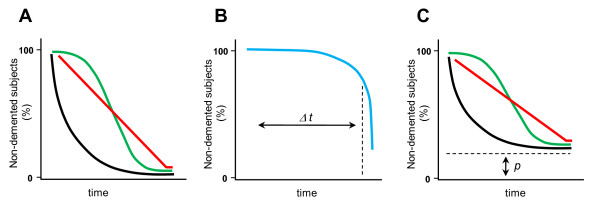
**Possible trajectories of the conversion process from cognitively normal to Alzheimer's disase (AD)-type dementia**. **(A) **Three different possible trajectories of the conversion to dementia in a group of cognitively normal (CN) individuals (100% of non-demented subjects at t_0_), at risk of AD. In the first trajectory (red line), the group comprises at baseline (t_0_) CN individuals at different stages of preclinical AD, with roughly the same number of subjects at each stage. The total conversion time (the time between appearance of an early AD marker and dementia onset) is constant and is the same for all subjects (t), and the number of converters in a given period is constant. In the second scenario (green line), the group comprises peole with preclinical AD, with a Gaussian distribution of the individuals at different stages of advancement (most individuals being at the intermediate stage). The total conversion time is constant and the same for all the individuals (t). Most of the group converts to dementia at around t_1/2_. Finally, the black line shows the group comprising CN at preclinical AD, with the constant conversion rate (proportion of the individuals that develop dementia in a given time period). Most individuals convert to dementia early, and the mean time of conversion is higher than the respective median. **(B) **Preclinical AD individuals with a passive compensatory mechanism that delays conversion for a given time (Δt), until the mechanism is exhausted. Subsequently, all patients convert to dementia, following one of the trajectories presented on the panel A. **(C) **Preclinical AD individuals with an active compensatory mechanism that prevents, in a certain proportion of cases (p), conversion to dementia, whichever trajectory the conversion process follows.

Different terms have been proposed to label these symptom-free individuals, who are positive for one or more early AD biomarkers. Most commonly, this phase of the disease has been called 'preclinical', 'presymptomatic', or 'asymptomatic' AD. In their recent recommendations, the National Institute on Ageing and the Alzheimer's Association workgroup have advocated the term 'preclinical' as the one that 'was felt to best encompass this conceptual phase of the disease' [167]. Even if these terms are still applied interchangeably, their use could reflect different viewpoints about the natural course of AD and the clinical significance of the early markers. Terms such as 'presymptomatic' or 'preclinical', in contrast to 'asymptomatic', imply that early markers not only indicate increased risk of AD-type dementia but that they precede and predict, at the individual level, clinical disease onset. It is now widely accepted that a morbid process that conveys transition from asymptomatic cerebral amyloidosis to AD-type dementia takes on average about 10 years [167]. There is indirect evidence in support of this point of view. At the population level, there is a lag of 10 years between the first detectable Aβ deposits (at autopsy) and dementia onset. In fact, the prevalence of CN people with Aβ deposits in their sixth decade of life is roughly the same as the prevalence of AD-type dementia one decade later [167]. However, such estimation is uncertain in the absence of definitive data on the dynamics of conversion to dementia of the CN population at risk of AD. Theoretically, various trajectories are possible. The conversion of CN to AD could be a linear process, with a steady cognitive decline and a constant number of converters over a given period [169]. In this case, the group at risk of AD would include CN individuals with a more or less advanced morbid process, which lasts a constant period of time. All of the CN individuals would convert to AD, and the more advanced the process in a given subject, the smaller the lag time to AD conversion. If the group comprised roughly the same number of individuals at each preclinical stage (1, 2 or 3), the process would be linear, but if the distribution of the different stages were Gaussian (most people being at the intermediate advanced stage), the conversion process would be better represented by a sigmoid curve (Figure 1A). Alternatively, conversion from preclinical AD to MCI/dementia could be determined by a purely stochastic process, with a constant percentage of individuals converting in a given period. This may correspond to a 'two-hits model', where the first hit (represented by the presence of a first preclinical AD marker) generates vulnerability, which increases at a constant rate the risk for a second hit and conversion to AD-type dementia. In this scenario, most people would convert to MCI/dementia early, and the median of the conversion time would be much shorter than with the linear or sigmoid models (Figure 1A). However, he recent data of Knopman and coworkers, showing a gradual increase in risk of conversion to MCI/dementia across the preclinical AD stages, do not support this possibility [168].

Independently of the dynamics involved, the conversion of the CN population at risk for AD to dementia may be influenced by compensatory mechanisms. Numerous data from both fMRI (for example, extensive extrahippocampal activation during memory activation tasks [157]) and biochemical studies (for example, increased choline acetyltransferase activity and the level of neurotrophic factors [170,171]) seem to support the idea that functional compensation is a major event in the course of AD. These compensatory mechanisms could be 'passive' or 'active'. A 'passive' compensatory process, referring to the notion of cognitive reserve, may only delay the conversion to dementia (Figure 1B). In agreement with this possibility, the cognitive decline preceding AD-type dementia fits a bi-logistic model with a plateau phase, and thus favorsthe idea of such compensation [172]. On the other hand, an 'active' and potentially inexhaustible compensatory mechanism could stop the progression of the disease at the preclinical phase, and prevent conversion to dementia. The efficiency of such active compensatory mechanisms is of key importance, as they may prevent the development of clinically overt dementia in some carriers of early AD marker(s) (Figure 1C).

The existence of effective compensatory mechanisms and the fate of cognitively intact individuals carrying an early AD marker is a matter of debate, and some authors believe that all individuals with an ongoing AD morbid process will inevitably progress to AD-type dementia if they were to live long enough [106,167]. In the absence of long-term longitudinal studies, the issue remains unresolved; however, certain lines of evidence challenge this idea. For instance, in an 8-year longitudinal study, Fagan and coworkers reported that only some CN older individuals with increased CSF tau/Aβ42 ratio converted to dementia [77]. Similarly, only a small number of CN individuals with increased PiB load converted to MCI or AD within 3 years [107]. Of course, it cannot be formally excluded that at least some of these CN individuals would eventually develop dementia if they were followed up for a sufficiently long period. However, the curve representing the conversion of CN individuals at risk for AD to dementia is strikingly biphasic. Some individuals convert to dementia rapidly within the first 3 years, whereas others remain cognitively stable over at least 8 years [77]. It is thus likely that some of the preclinical AD cases do not progress to dementia because they have efficient compensatory mechanisms. In line with this presumption, it has been shown that some CN subjects can maintain or even decrease their Aβ burden over time. Most interestingly, even those patients with high Aβ load, indistinguishable from the ones with AD-type dementia, can remain cognitively stable [95,107].

Several medical conditions share with AD the long clinical evolution and presymptomatic phase. It has been suggested that preclinical AD markers play a similar role in the early detection of AD as do increased blood glucose level or preclinical tumor markers in the early diagnosis of type II diabetes or cancer, respectively, for instance [167]. However, it needs to be kept in mind that in contrast to asymptomatic hyperglycemia or carcinoma *in situ*, which, if not treated, will inevitably progress to clinically overt disease, there is to date insufficient evidence to assert that preclinical AD imposes such determinism. Thus, any reliable predictions at the individual level on the basis of available preclinical AD markers are still very difficult. This, in turn, might raise important ethical concerns about disclosure of the information based on biomarker status and pre-AD state [173], especially in view of the current lack of curative treatments.

### Alzheimer disease-related neurodegeneration: *in vivo *indices of compensatory mechanisms

It is commonly believed that curative interventions in AD, especially those targeting Aβ, might be most effective when applied at the preclinical phase, because this precedes irreversible brain lesions [53,174]. However, the preclinical phase of AD could also be seen as a unique therapeutic window because at this stage the brain compensatory mechanisms are still efficient. Regardless of its exact molecular substrates, AD-type dementia may be viewed as a failure of these compensatory mechanisms in the course of progressive cerebral amyloidosis. One attractive scenario would be to treat AD not only by decreasing Aβ or tau brain load, but also by preserving these natural compensatory mechanisms. However such approaches remain purely speculative, as our understanding of the compensatory mechanisms is still very limited. Nevertheless, some evidence sustains the presence of active compensatory mechanisms in AD. For instance, there is a differential sensitivity of neurons to Aβ oligomers toxic effect. Although Aβ deposits are often localized in the striatum in both familial and sporadic AD cases, they are not associated with neuron loss in this brain region or with extrapyramidal symptoms [103,175]. Moreover, the *APOE ε3 *genotype, which in contrast to the *APOE ε4 *allele, decreases the risk of AD, has been shown to protect neurons from hyperexcitability [176,177], further supporting the notion that active neuroprotection plays an important role in cell vulnerability in AD.

## Conclusions

**Figure 2:**
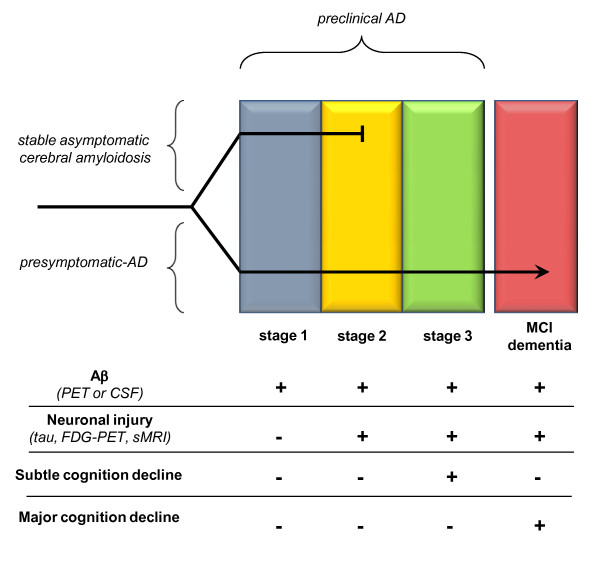
**Hypothetical model of preclinical Alzheimer's disease (AD)**. According to the proposed model, the group currently defined as 'preclinical AD' is heterogeneous and comprises two subpopulations. Firstly, there is the group of individuals at different stages of preclinical AD defined by the biomarkers indicated in the lower panel of the figure. All of these individuals will progress to dementia, and we call this phase 'presymptomatic AD'. The second group comprises individuals who are positive for amyloid markers and neuronal injury markers, and fall into one of the stages of preclinical AD, based on the current classification. However, this population has efficient active compensatory mechanisms, and remains resistant to dementia (stable asymptomatic cerebral amyloidosis).

Preclinical AD markers may represent a double-edged sword. On the one hand, they make it possible to define a group at risk for AD-type dementia (in terms of disease prevalence), but on the other hand, this group may comprise an increased proportion of 'resistant' individuals, who do not develop dementia despite substantial brain cerebral amyloidosis. Within the preclinical AD spectrum, the firstgroup includes presymptomatic individuals who are positive for at least one amyloid marker (for example,, PiB-PET, low Aβ42 CSF levels) and correspond to stages 1, 2 or 3 as defined by the recommendations from the National Institute on Aging and Alzheimer's Association workgroups [167]. Virtually all of these subjects will convert to MCI or AD-type dementia within 8 to 10 years. A second group includes individuals with stable asymptomatic cerebral amyloidosis, who will remain cognitively stable indefinitely, even though they have positive amyloid marker(s) and would fall into the stage 1 (or even stage 2) of preclinical AD (Figure 2). Defining distinct biomarkers for these stable cases would enable more reliable predictions of clinical evolution at the individual level. Moreover, comparative analysis of these two groups could allow better insight into the nature of compensatory mechanisms and into the reasons for their failure, which marks the beginning of AD-type dementia.

## Competing interests

The authors report no biomedical financial interests or potential conflicts of interest.

## Authors' contributions

MJL and PG performed the literature search, formulated the present hypothesis, and compiled the first draft of the manuscript. MJL created the figures. PRH and CB participated in the conceptualization and writing of the paper. All authors have read and approved the final manuscript.

## Pre-publication history

The pre-publication history for this paper can be accessed here:

http://www.biomedcentral.com/1741-7015/10/127/prepub
